# Clinical presentation and survival outcomes of well‐differentiated thyroid cancer in Filipinos

**DOI:** 10.1002/cam4.4149

**Published:** 2021-07-21

**Authors:** Uchechukwu C. Megwalu, Yifei Ma, Nosayaba Osazuwa‐Peters, Lisa A. Orloff

**Affiliations:** ^1^ Department of Otolaryngology—Head and Neck Surgery Stanford University School of Medicine Stanford California USA; ^2^ Department of Head and Neck Surgery and Communication Sciences Duke University School of Medicine Durham North Carolina USA

**Keywords:** Asians, Filipino, health status disparities, minority health, thyroid cancer

## Abstract

**Background:**

Filipinos have higher recurrence rates compared to other racial/ethnic groups, which might suggest a higher propensity for aggressive disease. The goal of this study was to perform a population‐based analysis of disease extent at diagnosis and survival outcomes in Filipino patients with well‐differentiated thyroid cancer relative to other racial/ethnic groups.

**Methods:**

The study cohort comprised adult patients with well‐differentiated thyroid cancer diagnosed between 2004 and 2015, identified in the California Cancer Registry. Rates of extrathyroidal extension, nodal metastasis, and distant metastasis were compared between Filipinos, Non‐Filipino Asians, and Non‐Asians using multilevel logistic regression models. Survival outcomes were compared using Cox regression models, utilizing a sequential modeling approach.

**Results:**

Filipino ethnicity was associated with extrathyroidal extension (OR 1.35, 95% CI 1.11–1.63) compared with non‐Asians and non‐Filipino Asians. Filipino ethnicity was also associated with nodal metastasis (OR 1.32, 95% CI 1.18–1.46), and with worse OS (Hazard Ratio [HR] 1.45, 95% CI 1.20–1.75) and DSS (HR 1.51, 95% CI 1.12–2.04). After adjusting for demographic and clinical factors, Filipino ethnicity was no longer associated with OS (HR 1.03, 95% CI 0.84–1.25) or DSS (HR 0.93, 95% CI 0.68–1.28).

**Conclusion:**

Filipino patients with thyroid cancer are more likely to present with locoregionally advanced disease compared with non‐Filipino Asians and non‐Asians. Furthermore, Filipino patients have worse survival outcomes compared with non‐Filipino Asians and non‐Asians. However, this appears to be driven by the higher rates of locoregionally advanced disease in Filipino patients.

## Graphical abstract

Filipino patients with thyroid cancer are more likely to present with locoregionally advanced disease compared with non‐Filipino Asians and non‐Asians. This results in worse survival outcomes for Filipino patients.

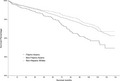

## 1 INTRODUCTION

The incidence of thyroid cancer is rapidly increasing in the United States (US).^1^ The incidence increased from 4.56 per 100,000 person‐years in 1974–1977 to 14.42 per 100,000 person‐years in 2010–2013.^2^, ^3^, ^4^, ^5^ Although thyroid cancer is associated with low mortality rates,^6^ significant racial disparities exist in thyroid cancer outcomes. African American patients present with more advanced tumors, are less likely to receive appropriate treatment, and have worse survival outcomes than other racial groups, even after adjusting for disease stage and other clinically important factors.^7^, ^8^, ^9^, ^10^, ^11^ While there is extensive evidence of poor outcomes in African Americans, racial disparities in outcomes have not been adequately studied for Asians, even though they make up a significant proportion of the US population. Furthermore, the Asian population comprises diverse ethnic groups that are not homogenous.

The incidence of thyroid cancer is high in Southeast and East Asians. Among these, Filipinos have one of the highest incidence rates in the world.^12^, ^13^ Filipino patients with thyroid cancer have higher rates of recurrence compared to other racial/ethnic groups, which might suggest a higher propensity for aggressive disease.^14^ A recent evaluation of US national mortality records indicated that age‐adjusted mortality rates due to thyroid cancer were highest in Filipinos.^15^ However, it is not clear if this is due to the overall higher incidence of thyroid cancer in this population, or due to worse survival outcomes compared to other racial/ethnic groups. The goal of this study was to perform a population‐based analysis of disease extent at diagnosis and survival outcomes in Filipino patients with well‐differentiated thyroid cancer relative to other racial/ethnic groups. We hypothesized that Filipino patients have higher rates of advanced stage disease, and consequently, worse survival compared with non‐Filipino Asians and non‐Asians.

## 2 MATERIALS AND METHODS

This study was approved by the State of California Committee for the Protection of Human Subjects, and was considered exempt by the Stanford University Institutional Review Board. Data were extracted from the California Cancer Registry (CCR) dataset. The CCR is a statewide population‐based cancer surveillance system that also comprises three SEER registries.^16^ It is the largest, contiguous‐area, population‐based cancer registry system in the country, and has been collecting data under state mandate using uniform, high‐quality reporting standards since 1988. The study cohort comprised adult patients with papillary thyroid carcinoma or follicular/Hurthle cell carcinoma diagnosed between 1 January 2004 and 31 December 2015.

Tumor site and histology were determined according to the ICD‐O‐3 topography (C73.9 for thyroid gland) and morphology codes available in CCR.^17^ The following codes for papillary, follicular, and Hurthle cell carcinoma were included: 8050/3, 8260/3, 8340/3, 8342/3, 8343/3, 8344/3, 8330/3, 8331/3, 8332/3, and 8335/3. Information on disease stage was obtained from CCR using the SEER‐AJCC stage. This is based on combined clinical and pathological assessments, including all information available within 4 months of diagnosis or through completion of surgery in the first course of treatment. Staging information is based on (in priority order) the pathology report, the surgeon's evaluation at the time of surgery, clinical findings, or radiographic description.^18^ Patients were classified according to the AJCC staging 6th edition^19^ for patient diagnosed during 2004–2009, and 7th edition^20^ for patients diagnosed after 2009.

Race/ethnicity was categorized as Filipino, Non‐Filipino Asian, or Non‐Asian. Marital status was categorized as “married” (including common law) or “single” (single‐never married, divorced, and widowed). Insurance status was categorized as commercial, Medicare, Medicaid, uninsured, or unknown. Neighborhood‐level socioeconomic status (SES) was classified into five quintiles, lowest (SES‐1), lower‐middle (SES‐2), middle (SES‐3), higher‐middle (SES‐4), and highest (SES‐5) based on the Yost score. The Yost score is a composite index of SES contained in the CCR that is based on principal component analysis of block group level census variables such as education, income, and occupation.^21^

The statistical analysis was performed using SAS system, version 9.4 (SAS Institute, Inc.,). Unadjusted and adjusted logistic regression models were used to assess the association between race/ethnicity and the following measures for extent of disease: extrathyroidal extension, nodal metastasis, and distant metastasis. Unadjusted and adjusted multilevel linear regression models were used to assess the association between race/ethnicity and tumor size. For the linear regression models, tumor size was log transformed to improve model fit because the distribution of tumor size was right skewed. For all adjusted analyses assessing the association between race/ethnicity and extent of disease, the following covariates were entered a priori into the model: age, marital status, sex, Charlson comorbidity score, SES, and insurance status. The association between race/ethnicity and overall survival (OS) and disease‐specific survival (DSS) was assessed using Kaplan–Meier survival analysis, and unadjusted and adjusted Cox regression models with robust standard error using sandwich variance estimators. Assumption of proportionality in hazards was evaluated and satisfied. In order to understand the drivers of the racial/ethnic disparities in survival, a sequential modeling approach was applied for the adjusted models. Each stage included the following predictors sequentially: (1) demographic (age, sex, and marital status) and clinical factors (T classification, N classification, M classification, Charlson comorbidity score, histology, and extent of thyroidectomy); (2) neighborhood SES; and (3) insurance status. Cases with missing values were excluded from analysis. An estimate was considered to be statistically significant at α = 0.05.

## 3 RESULTS

We identified 36,573 patients meeting the inclusion criteria: 2065 Filipinos, 4327 non‐Filipino Asians, and 30,181 Non‐Asians. Patient characteristics are shown in Table 1. Filipino patients were older than non‐Filipino Asians and non‐Asians at diagnosis (51.3 years vs. 47.3 years and 47.7 years, respectively). Slightly higher proportion of non‐Asians (22.3%) were male, compared with Filipino Asians (20.5%) and non‐Filipino Asians (20.7%).

**TABLE 1:** Patient characteristics

	Filipino	Non‐Filipino Asians	Non‐Asians	*p* value
Variable	Mean (SD)	Mean (SD)	Mean (SD)	
Age	51.3 (14.6)	47.3 (14.8)	47.7 (14.9)	<0.001
Tumor size (cm)	2.1 (1.7)	2.0 (1.7)	2.0 (1.9)	0.03

Variable	Number (%)	Number (%)	Number (%)	
Male	423 (20.5%)	895 (20.7%)	6742 (22.3%)	0.01
Female	1642 (79.5%)	3432 (79.3%)	23439 (77.7%)	
Married	1393 (70.2%)	3015 (71.8%)	18117 (62.0%)	<0.001
Single	592 (29.8%)	1182 (28.2%)	11094 (38.0%)	
Insurance: Commercial	1420 (68.8%)	3238 (74.8%)	21720 (72.0%)	<0.001
Insurance: Medicare	149 (7.2%)	277 (6.4%)	2809 (9.3%)	
Insurance: Medicaid	432 (20.9%)	668 (15.4%)	4607 (15.3%)	
Insurance: Uninsured	41 (2.0%)	94 (2.2%)	651 (2.2%)	
Insurance: Unknown	23 (1.1%)	50 (1.2%)	394 (1.3%)	
SES quintile 1	182 (8.8%)	332 (7.7%)	4397 (14.6%)	<0.001
SES quintile 2	375 (18.2%)	617 (14.3%)	5603 (18.6%)	
SES quintile 3	488 (23.6%)	796 (18.4%)	6164 (20.4%)	
SES quintile 4	609 (29.5%)	1111 (25.7%)	6776 (22.5%)	
SES quintile 5	411 (19.9%)	1471 (34.0%)	7241 (24.0%)	
Charlson score: 0	1161 (71.4%)	2874 (83.5%)	19597 (76.8%)	<0.001
Charlson score: 1	315 (19.4%)	406 (11.8%)	4264 (16.7%)	
Charlson score: 2	79 (4.9%)	86 (2.5%)	980 (3.8%)	
Charlson score: 3+	72 (4.4%)	74 (2.2%)	674 (2.6%)	
T classification: T1	956 (48.1%)	2221 (53.5%)	16040 (54.9%)	<0.001
T classification: T2	397 (20.0%)	721 (17.4%)	5536 (18.9%)	
T classification: T3	491 (24.7%)	1006 (24.2%)	6302 (21.6%)	
T classification: T4	142 (7.2%)	207 (5.0%)	1364 (4.7%)	
N classification: N1	614 (30.5%)	1249 (29.8%)	8001 (27.0%)	<0.001
N classification: N0	1396 (69.5%)	2940 (70.2%)	21609 (73.0%)	
M classification: M1	60 (3.0%)	95 (2.3%)	498 (1.7%)	<0.001
M classification: M0	1970 (97.0%)	4124 (97.7%)	29213 (98.3%)	
AJCC stage I	1215 (62.0%)	2923 (70.9%)	21002 (72.1%)	<0.001
AJCC stage II	166 (8.5%)	251 (6.1%)	2255 (7.7%)	
AJCC stage III	327 (16.7%)	578 (14.0%)	3613 (12.4%)	
AJCC stage IV	251 (12.8%)	371 (9.0%)	2267 (7.8%)	
Papillary thyroid carcinoma	1949 (94.4%)	4116 (95.1%)	28434 (94.2%)	0.05
Follicular thyroid carcinoma	116 (5.6%)	211 (4.9%)	1747 (5.8%)	

### 3.1 Extent of disease at diagnosis

The association between race/ethnicity and extent of local disease is shown in Table 2. On univariable analysis, Filipino patients had larger tumor size (difference 1.2%, 95% confidence interval [CI] 0.6%–1.8%) and higher odds of extrathyroidal extension (odds ratio [OR] 1.54, 95% CI 1.28–1.85) compared to non‐Asians. There was no difference in tumor size (difference 0.5%, 95% CI 0.0%–0.9%) or rates of extrathyroidal extension (OR 1.08, 95% CI 0.93–1.26) between non‐Filipino Asians and non‐Asians. On multivariable analysis, Filipino ethnicity remained associated with increased tumor size (difference 8.1%, 95% CI 3.5%–12.9%) and extrathyroidal extension (OR 1.35, 95% CI 1.11–1.63) after adjusting for age, marital status, sex, Charlson comorbidity score, SES, and insurance status. Non‐Filipino Asian ethnicity was associated with increased tumor size (difference 3.3%, 95% CI 0.1%–6.6%), but not extrathyroidal extension (OR 1.14, 95% CI 0.97–1.34).

**TABLE 2:** Association between Filipino ethnicity and tumor size and extrathyroidal extension

	Tumor size			Extrathyroidal extension		
Variable	% Difference	95% CI	*p* value	Odds ratio	95% CI	*p* value
**Unadjusted**						
Filipino	1.2%	0.6%–1.8%	<0.001	1.54	1.28–1.85	<0.001
Non‐Filipino Asians	0.5%	0.0%–0.9%	0.04	1.08	0.93–1.26	0.32
Non‐Asians	0.0%		Reference	1.00		Reference
**Adjusted**						
Filipino	8.1%	3.5%–12.9%	<0.001	1.35	1.11–1.63	0.002
Non‐Filipino Asians	3.3%	0.1%–6.6%	0.04	1.14	0.97–1.34	0.11
Non‐Asians	0.0%		Reference	1.00		Reference
Age (10‐year increments)	−6.3%	−7.0% to −5.6%	<0.001	1.37	1.31–1.42	<0.001
Married	−5.8%	−7.7% to −3.8%	<0.001	0.92	0.83–1.02	0.12
Single	0.0%		Reference	1.00		Reference
Male	35.7%	32.5%–39.0%	<0.001	1.73	1.55–1.93	<0.001
Female	0.0%		Reference	1.00		Reference
Charlson score: 0	0.0%		Reference	1.00		Reference
Charlson score: 1	−0.4%	−3.25% to 2.6%	0.80	1.22	1.06–1.40	0.005
Charlson score: 2	−6.5%	−11.8% to −1.0%	0.02	1.34	1.07–1.67	0.01
Charlson score: 3+	5.5%	−1.5% to 13.1%	0.13	1.14	0.88–1.46	0.32
Charlson score: Unknown	−0.1%	−3.0% to 2.9%	0.96	0.83	0.71–0.97	0.02
SES quintile 1	0.0%		Reference	1.00		Reference
SES quintile 2	−4.2%	−7.6% to −0.7%	0.02	0.90	0.76–1.06	0.20
SES quintile 3	−6.9%	−10.1% to −3.5%	<0.001	0.78	0.66–0.92	0.003
SES quintile 4	−9.5%	−12.7% to −6.2%	<0.001	0.73	0.62–0.87	<0.001
SES quintile 5	−12.0%	−15.2% to −8.7%	<0.001	0.59	0.49–0.71	<0.001
Insurance: Commercial	0.0%		Reference	1.00		Reference
Insurance: Uninsured	13.4%	5.2%–22.2%	0.001	1.62	1.17–2.24	0.004
Insurance: Medicare	10.5%	6.2%–14.9%	<0.001	1.26	1.07–1.49	0.005
Insurance: Medicaid	15.5%	11.8%–19.2%	<0.001	1.59	1.38–1.84	<0.001
Insurance: Unknown	7.2%	−2.2% to 17.5%	0.14	1.58	1.05–2.40	0.03

The associations between race/ethnicity and nodal and distant metastasis are shown in Table 3. On univariable analysis, Filipino patients had higher likelihood of nodal metastasis (OR 1.16, 95% CI 1.05–1.28), while non‐Filipino Asians had similar likelihood of nodal metastasis (OR 1.06, 95% CI 0.98–1.14) to non‐Asians. On multivariable analysis, Filipino ethnicity remained associated with nodal metastasis (OR 1.32, 95% CI 1.18–1.46). Non‐Filipino Asian ethnicity was also associated with nodal metastasis, but to a lesser degree (OR 1.09, 95% CI 1.01–1.18). On univariable analysis, Filipino (OR 1.69, 95% CI 1.28–2.23) and non‐Filipino Asian (OR 1.28, 95% CI 1.02–1.61) ethnicities were associated with distant metastasis. On multivariable analysis, non‐Filipino Asian ethnicity remained associated with distant metastasis (OR 1.42, 95% CI 1.11–1.80). However, Filipino ethnicity was no longer associated with distant metastasis (OR 1.32, 95% CI 0.98–1.78).

**TABLE 3:** Association between Filipino ethnicity and nodal and distant metastasis

	Nodal metastasis			Distant metastasis		
Variable	Odds ratio	95% CI	*p* value	Odds ratio	95% CI	*p* value
**Unadjusted**						
Filipino	1.16	1.05–1.28	0.004	1.69	1.28–2.23	<0.001
Non‐Filipino Asians	1.06	0.98–1.14	0.13	1.28	1.02–1.61	0.03
Non‐Asians	1.00		Reference	1.00		Reference
**Adjusted**						
Filipino	1.32	1.18–1.46	<0.001	1.32	0.98–1.78	0.06
Non‐Filipino Asians	1.09	1.01–1.18	0.03	1.42	1.11–1.80	0.004
Non‐Asians	1.00		Reference	1.00		Reference
Age (10‐year increments)	0.76	0.74–0.77	<0.001	1.63	1.54–1.74	<0.001
Married	0.92	0.87–0.97	0.002	0.68	0.58–0.81	<0.001
Single	1.00		Reference	1.00		Reference
Male	1.91	1.80–2.02	<0.001	2.45	2.07–2.90	<0.001
Female	1.00		Reference	1.00		Reference
Charlson score: 0	1.00		Reference	1.00		Reference
Charlson score: 1	1.12	1.04–1.20	0.003	1.32	1.06–1.64	0.01
Charlson score: 2	1.02	0.88–1.19	0.75	1.73	1.27–2.35	0.001
Charlson score: 3+	1.36	1.15–1.62	<0.001	2.09	1.54–2.82	<0.001
Charlson score: Unknown	0.99	0.92–1.06	0.76	0.97	0.76–1.25	0.83
SES quintile 1	1.00		Reference	1.00		Reference
SES quintile 2	0.98	0.90–1.07	0.65	1.15	0.88–1.49	0.30
SES quintile 3	0.90	0.82–0.98	0.02	0.92	0.70–1.21	0.55
SES quintile 4	0.85	0.77–0.92	<0.001	0.85	0.65–1.12	0.26
SES quintile 5	0.89	0.81–0.98	0.01	0.67	0.50–0.90	0.009
Insurance: Commercial	1.00		Reference	1.00		Reference
Insurance: Uninsured	1.08	0.90–1.29	0.40	1.96	1.13–3.37	0.02
Insurance: Medicare	1.19	1.07–1.32	0.001	1.26	0.99–1.61	0.06
Insurance: Medicaid	1.12	1.03–1.21	0.005	2.07	1.66–2.57	<0.001
Insurance: Unknown	1.20	0.97–1.50	0.10	1.97	1.02–3.81	0.04

### 3.2 Survival outcomes

**FIGURE 1:**
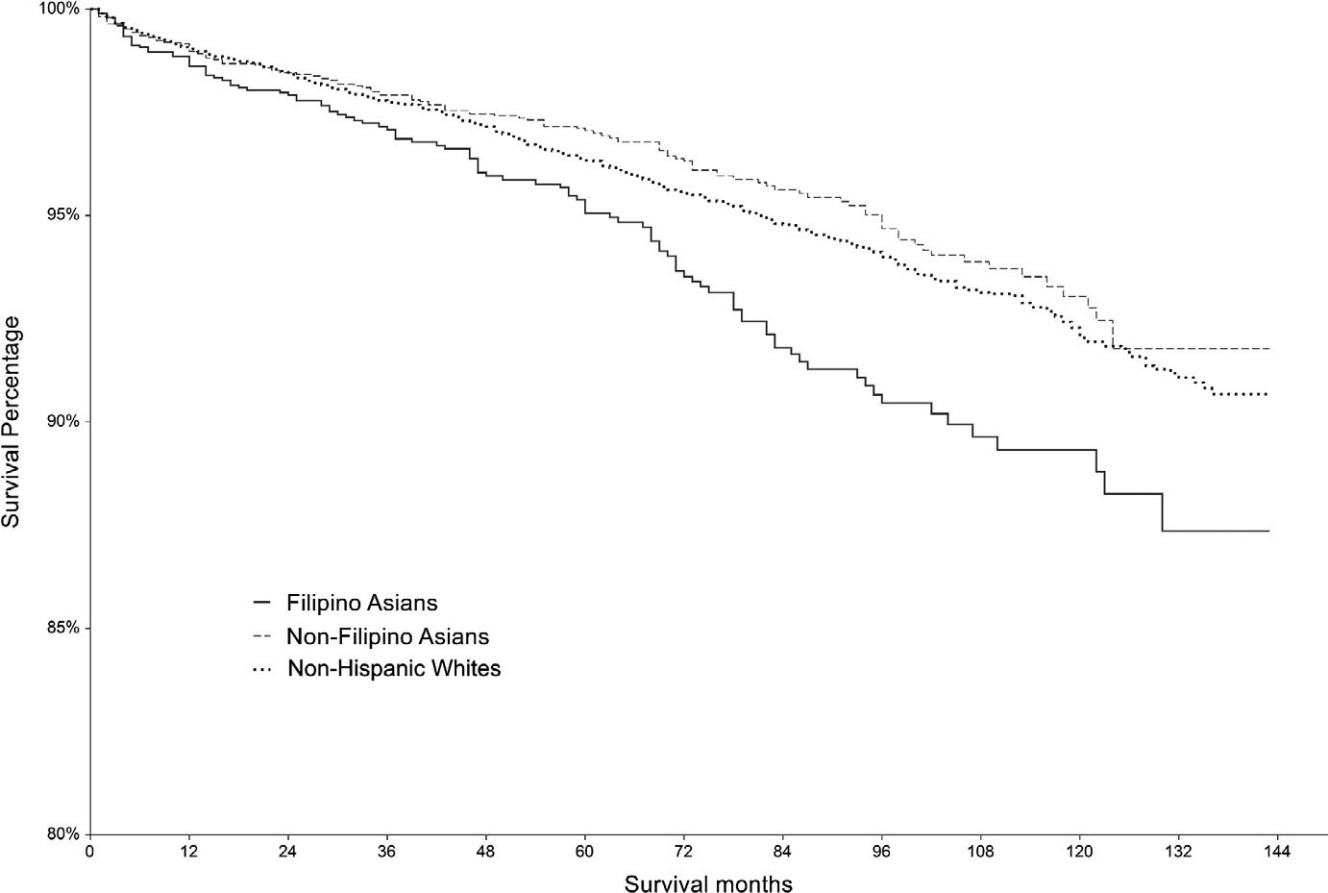
Association between Filipino ethnicity and overall survival

**FIGURE 2:**
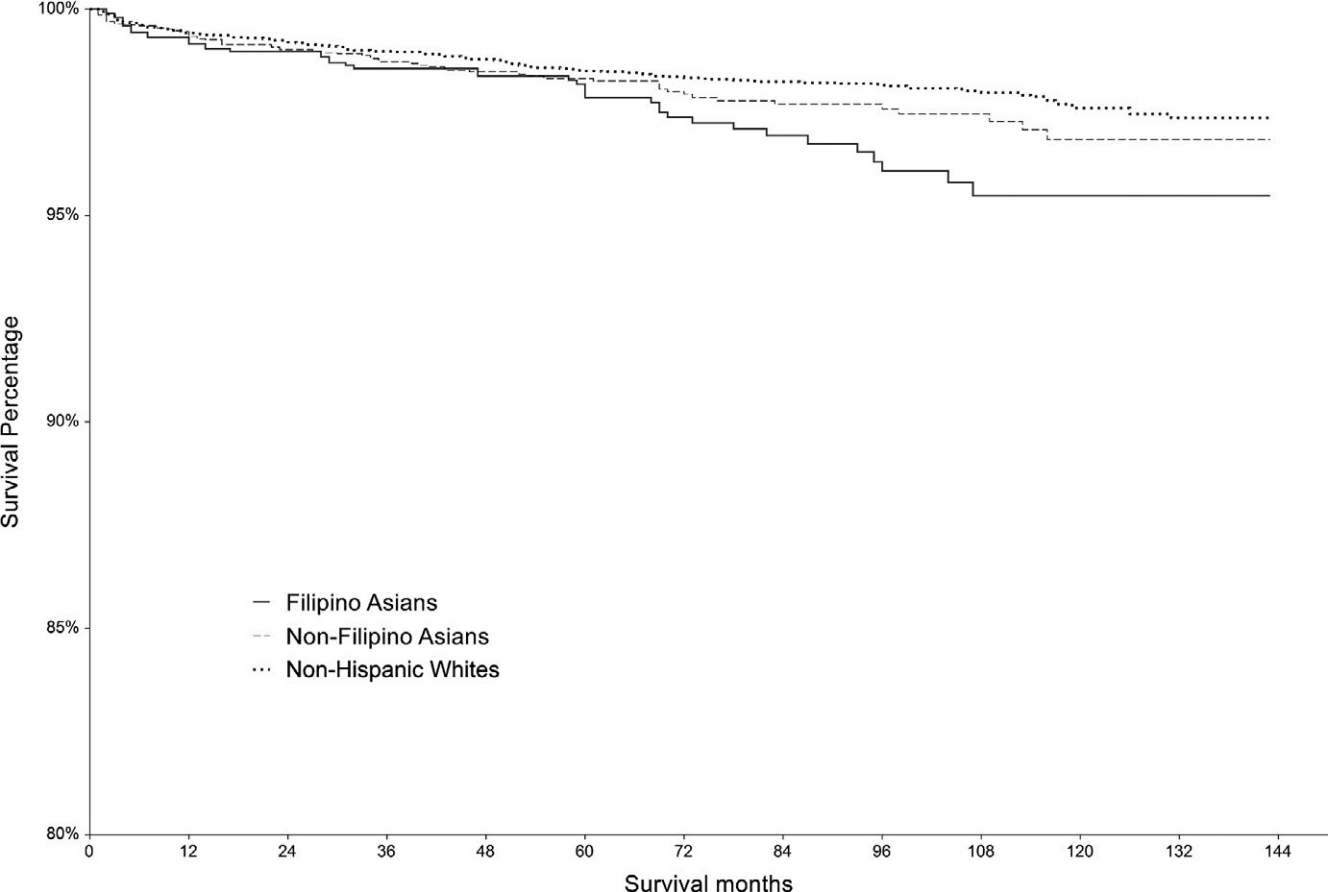
Association between Filipino ethnicity and disease‐specific survival

Filipino patients had worse OS (5‐year OS 95.1%, 95% CI 93.9%–96.2%) than non‐Filipino Asians (5‐year OS 97.1%, 95% CI 96.5%–97.7%) and non‐Asians (5‐year OS 96.3%, 95% CI 96.0%–96.7%, *p* < 0.001) (Figure 1). Filipino patients had worse DSS (5‐year DSS 97.9%, 95% CI 97.1%–98.6%) than non‐Filipino Asians (5‐year DSS 98.3%, 95% CI 97.9%–98.8%) and non‐Asians (5‐year DSS 98.4%, 95% CI 98.2%–98.6%, *p* = 0.02) (Figure 2). The results of sequential modeling analysis evaluating the association between race/ethnicity and survival are shown in Table 4. Unadjusted analysis shows that Filipino ethnicity is associated with worse OS (Hazard Ratio [HR] 1.45, 95% CI 1.20–1.75) and DSS (HR 1.51, 95% CI 1.12–2.04) compared with non‐Asians. Non‐Filipino Asian ethnicity was not associated with OS (HR 0.86, 95% CI 0.72–1.02) or DSS (HR 1.12, 95% CI 0.87–1.44). After adjusting for demographic and clinical factors, Filipino ethnicity was no longer associated with OS (HR 1.03, 95% CI 0.84–1.25) or DSS (HR 0.93, 95% CI 0.68–1.28). However, non‐Filipino Asian ethnicity was associated with improved OS (HR 0.79, 95% CI 0.64–0.96), but not with DSS (HR 0.93, 95% CI 0.71–1.22). In the final model, adjusting for demographic factors, clinical factors, SES, and insurance status, Filipino ethnicity was not associated with OS (HR 1.01, 95% CI 0.82–1.24) or DSS (HR 0.91, 95% CI 0.66–1.26). In the final model, non‐Filipino Asian ethnicity remained associated with improved OS (HR 0.78, 95% CI 0.64–0.95), but not with DSS (HR 0.91, 95% CI 0.68–1.21).

**TABLE 4:** Association between Filipino ethnicity and survival (sequential modeling)

	Overall survival			Disease‐specific survival		
Variable	Hazard ratio	95% CI	*p* value	Hazard ratio	95% CI	*p* value
**Unadjusted**						
Filipino	1.45	1.20–1.75	<0.001	1.51	1.12–2.04	0.007
Non‐Filipino Asians	0.86	0.72–1.02	0.09	1.12	0.87–1.44	0.36
Non‐Asians	1.00		Reference	1.00		Reference
**Adjusted for demographic and clinical factors**						
Filipino	1.03	0.84–1.25	0.80	0.93	0.68–1.28	0.66
Non‐Filipino Asians	0.79	0.64–0.96	0.02	0.93	0.71–1.22	0.60
Non‐Asians	1.00		Reference	1.00		Reference
**Adjusted for demographic factors, clinical factors, and socioeconomic status**						
Filipino	1.06	0.87–1.30	0.54	0.94	0.68–1.31	0.73
Non‐Filipino Asians	0.81	0.66–0.99	0.04	0.95	0.72–1.25	0.70
Non‐Asians	1.00		Reference	1.00		Reference
**Adjusted for demographic factors, clinical factors, socioeconomic status, and insurance status**						
Filipino	1.01	0.82–1.24	0.93	0.91	0.66–1.26	0.57
Non‐Filipino Asians	0.78	0.64–0.95	0.01	0.91	0.68–1.21	0.50
Non‐Asians	1.00		Reference	1.00		Reference
Age (10‐year increments)	1.96	1.86–2.06	<0.001	1.91	1.78–2.06	<0.001
Male	1.63	1.44–1.86	<0.001	1.26	1.03–1.55	0.02
Female	1.00		Reference	1.00		Reference
Married	0.71	0.63–0.80	<0.001	0.90	0.74–1.11	0.33
Single	1.00		Reference	1.00		Reference
T classification: T1	1.00		Reference	1.00		Reference
T classification: T2	1.11	0.92–1.33	0.30	2.82	1.81–4.41	<0.001
T classification: T3	1.57	1.35–1.83	<0.001	5.71	3.89–8.39	<0.001
T classification: T4	4.18	3.50–4.99	<0.001	24.63	16.38–37.05	<0.001
T classification: Unknown	1.87	1.41–2.48	<0.001	7.35	4.62–11.67	<0.001
N classification: N0	1.00		Reference	1.00		Reference
N classification: N1	1.60	1.39–1.85	<0.001	1.95	1.55–2.47	<0.001
N classification: Unknown	1.58	1.21–2.08	0.001	1.48	1.02–2.17	0.04
M classification: M0	1.00		Reference	1.00		Reference
M classification: M1	3.44	2.82–4.19	<0.001	5.90	4.59–7.59	<0.001
M classification: Unknown	1.49	1.08–2.05	0.02	1.73	1.08–2.76	0.02
Charlson score: 0	1.00		Reference	1.00		Reference
Charlson score: 1	1.58	1.36–1.82	<0.001	1.31	1.03–1.66	0.03
Charlson score: 2	2.68	2.21–3.25	<0.001	1.66	1.20–2.29	0.002
Charlson score: 3+	3.82	3.10–4.71	<0.001	1.54	1.09–2.19	0.01
Charlson score: Unknown	1.09	0.89–1.33	0.41	0.74	0.53–1.04	0.09
Papillary thyroid carcinoma	1.00		Reference	1.00		Reference
Follicular thyroid carcinoma	1.22	1.00–1.48	0.05	1.41	1.05–1.88	0.02
Thyroid lobectomy	0.90	0.65–1.25	0.53	0.77	0.45–1.30	0.33
Total thyroidectomy	0.56	0.48–0.64	<0.001	0.46	0.36–0.58	<0.001
SES quintile 1	1.00		Reference	1.00		Reference
SES quintile 2	0.93	0.78–1.12	0.44	0.92	0.69–1.24	0.59
SES quintile 3	0.73	0.59–0.90	0.003	0.79	0.58–1.08	0.14
SES quintile 4	0.79	0.65–0.96	0.02	0.92	0.68–1.26	0.61
SES quintile 5	0.77	0.64–0.94	0.009	0.80	0.58–1.11	0.18
Insurance: Commercial	1.00		Reference	1.00		Reference
Insurance: Uninsured	1.03	0.63–1.69	0.91	1.08	0.55–2.12	0.82
Insurance: Medicare	1.20	1.03–1.41	0.02	0.84	0.65–1.09	0.19
Insurance: Medicaid	1.47	1.25–1.72	<0.001	1.19	0.92–1.54	0.19
Insurance: Unknown	1.55	1.02–2.34	0.04	1.80	0.89–3.64	0.10

Demographic factors: age, sex, and marital status.

Clinical factors: T classification, N classification, M classification, Charlson comorbidity score, histology, and extent of thyroidectomy.

## 4 DISCUSSION

Our study shows that Filipino patients with thyroid cancer are more likely to present with locoregionally advanced disease compared with non‐Filipino Asians and non‐Asians. Filipino ethnicity was associated with higher likelihood of extrathyroidal extension, and nodal metastasis, but not distant metastasis. Filipino patients had worse OS and DSS. However, this association disappeared after adjusting for demographic and clinical variables, including extent of disease, indicating that the difference in survival was due to extent of disease.

The findings are important because Filipinos have one of the highest incidence rates of thyroid cancer in the world,^12^, ^13^ and the highest incidence‐based thyroid cancer mortality in the United States.^15^ This is the largest study to assess the association between Filipino ethnicity and extant of disease and survival outcomes in patients with thyroid cancer. Only one previous study has evaluated the disease extent and outcomes in Filipinos relative to other racial/ethnic groups. Kus et al. performed a single‐institution retrospective study comparing thyroid cancer outcomes between 36 Filipino and 463 non‐Filipino patients.^14^ In contrast to our study, they found no difference in tumor size and disease stage between the two groups. There was also no significant difference in survival on unadjusted and adjusted analyses. However, Filipino patients had higher risk of recurrence. In contrast, our study found that Filipino patients had worse survival than non‐Filipino Asians and non‐Asians, but the survival difference disappeared after adjusting for disease stage, treatment factors, and comorbidity. The difference in findings between studies may be due to the small sample size of the study by Kus et al. Furthermore, it is unclear what variables were adjusted for in their analysis. Nguyen et al. studied thyroid cancer mortality in Filipino, non‐Filipino Asian, and non‐Hispanic White adults using US death records (2003–2012) and US Census data.^15^ They found that Filipinos had the highest age‐adjusted mortality rates due to thyroid cancer compared with non‐Filipino Asians and non‐Hispanic Whites. Since this study used data based on national death certificates, it is unclear if the high incidence‐based thyroid cancer mortality for Filipinos is due to the overall higher incidence of thyroid cancer in this population, or due to an actual difference in the disease process in this group relative to other racial/ethnic groups.

Several causes for the higher thyroid cancer mortality rates and recurrence in Filipinos have been suggested. Previous studies have theorized that exposure to carcinogenic volcanic lava, and interference of a high‐iodine diet with radioactive iodine treatment may contribute to worse outcomes.^15^, ^22^ However, our findings suggest that the worse survival outcomes observed in Filipinos is due to more locoregionally advanced disease in this population. The reasons for this difference in disease presentation remains unclear. The higher risk of locoregionally advanced disease in Filipinos remained, even after adjusting for age, sex, marital status, SES, and insurance status, indicating that these are not the drivers of this disparity. Consequently, the more advanced disease at present is unlikely to be due to difficulty accessing timely care. There may be a molecular basis for differences in extent of disease between Filipinos and non‐Filipinos. One possible explanation is that Filipinos may be predisposed to tumors that contain genetic mutations that lead to more locoregionally aggressive disease. Several small studies have examined the rates of BRAF V600E mutation (which is associated with more aggressive disease) in Filipino patients with mixed results. One of the studies reported higher rates of BRAF V600E mutation in Filipinos, while the other reported lower rates compared with reported rates for non‐Filipino Asians.^23^, ^24^ More robust studies are needed to determine whether there is a molecular cause that leads to more aggressive tumors in Filipinos relative to other racial/ethnic groups.

The results of our study suggest that clinicians should have a high index of suspicious for locoregionally advanced disease when managing Filipino patients with thyroid cancer. The higher rates of extrathyroidal extension and lymph node metastasis, and the resulting worse survival, raise the question of whether these patients should be managed differently from other racial/ethnic groups. Kus et al. recommended total thyroidectomy for Filipino patients with thyroid nodules 1 cm or larger, regardless of fine needle aspiration cytology findings.^14^ They also recommended more frequent follow‐up with imaging and serum thyroglobulin measurements for Filipino patients with thyroid cancer. However, there is currently no data on whether such aggressive management would translate to improved survival or reduced recurrence rates. Furthermore, this approach would most likely lead to unnecessary operations for benign thyroid nodules. Another pertinent question is whether this population may benefit from routine cross‐sectional imaging of the neck (computed tomography or magnetic resonance imaging), in order to detect extrathyroidal extension and lymph node metastasis that may not be readily apparent on examination. However, there is currently no evidence to support routine cross‐sectional imaging in this patient population.

The main strength of our study is its large sample size and diverse patient characteristics. The CCR is the largest, contiguous‐area, population‐based cancer registry system in the country, and has been collecting data under state mandate using uniform, high‐quality reporting standards since 1988.^16^ Since cancer reporting is mandated by California law, CCR data are representative of all cancer cases in California. Our study has several limitations. CCR does not provide information on disease recurrence, therefore, the association between Filipino ethnicity and disease recurrence could not be assessed. Also, this study was limited to patients treated in California. Therefore, it is unclear if the findings are generalizable to the entire United States. However, California has the highest population of Filipinos in the United States, with 45% of the nearly 3 million Filipinos in the United States living in California.^25^ Consequently, our findings are likely generalizable to the rest of the United States.

## 5 CONCLUSION

Filipino patients with thyroid cancer are more likely to present with locoregionally advanced disease compared with non‐Filipino Asians and non‐Asians. Furthermore, Filipino patients have worse survival outcomes compared with non‐Filipino Asians and non‐Asians. However, this appears to be driven by the higher rates of locoregionally advanced disease in Filipino patients. Further investigation is needed to determine whether there is a molecular basis for the higher propensity of Filipinos to present with locoregionally advanced disease. Furthermore, future studies are needed to determine whether this patient population would benefit from more aggressive work‐up and treatment.

## CONFLICT OF INTEREST

No competing financial interests exist.

## ETHICAL APPROVAL

This study was approved by the State of California Committee for the Protection of Human Subjects, and was considered exempt by the Stanford University Institutional Review Board.

## Data Availability

Research data are not shared due to California Cancer Registry policy.

## References

[1] American Cancer Society . Cancer facts & figures 2017. Accessed October 19, 2017. https://www.cancer.org/research/cancer‐facts‐statistics/all‐cancer‐facts‐figures/cancer‐facts‐figures‐2017.html

[2] Kilfoy BA, Zheng T, Holford TR, et al. International patterns and trends in thyroid cancer incidence, 1973–2002. Cancer Causes Control CCC. 2009;20(5):525‐531. 10.1007/s10552-008-9260-4 19016336 PMC2788231

[3] Davies L, Welch HG. Increasing incidence of thyroid cancer in the United States, 1973–2002. JAMA. 2006;295(18):2164‐2167. 10.1001/jama.295.18.2164 16684987

[4] Davies L, Welch HG. Current thyroid cancer trends in the United States. JAMA Otolaryngol Head Neck Surg. 2014;140(4):317‐322. 10.1001/jamaoto.2014.1 24557566

[5] Lim H, Devesa SS, Sosa JA, Check D, Kitahara CM. Trends in thyroid cancer incidence and mortality in the United States, 1974‐2013. JAMA. 2017;317(13):1974‐2013. 10.1001/jama.2017.2719 PMC8216772 28362912

[6] Li M, Brito JP, Vaccarella S. Long‐term declines of thyroid cancer mortality: an international age‐period‐cohort analysis. Thyroid Off J Am Thyroid Assoc. 2020;30(6):838‐846. 10.1089/thy.2019.0684 31964280

[7] Megwalu UC, Saini AT. Racial disparities in papillary thyroid microcarcinoma survival. J Laryngol Otol. 2017;131(1):83‐87. 10.1017/S0022215116009737 27917722

[8] Shah SA, Adam MA, Thomas SM, et al. Racial disparities in differentiated thyroid cancer: have we bridged the gap? Thyroid. 2017;27(6):762‐772. 10.1089/thy.2016.0626 28294040

[9] Keegan THM, Grogan RH, Parsons HM, et al. Sociodemographic disparities in differentiated thyroid cancer survival among adolescents and young adults in California. Thyroid. 2015;25(6):635‐648. 10.1089/thy.2015.0021 25778795 PMC4490589

[10] Harari A, Li N, Yeh MW. Racial and socioeconomic disparities in presentation and outcomes of well‐differentiated thyroid cancer. J Clin Endocrinol Metab. 2014;99(1):133‐141. 10.1210/jc.2013-2781 24243631 PMC3879674

[11] Hollenbeak CS, Wang L, Schneider P, Goldenberg D. Outcomes of thyroid cancer in African Americans. Ethn Dis. 2011;21(2):210‐215. 21749026

[12] Kolonel LN. Cancer incidence among Filipinos in Hawaii and the Philippines. Natl Cancer Inst Monogr. 1985;69:93‐98. 3834352

[13] Goodman MT, Yoshizawa CN, Kolonel LN. Descriptive epidemiology of thyroid cancer in Hawaii. Cancer. 1988;61(6):1272‐1281. 10.1002/1097-0142(19880315)61:6<1272:aid-cncr2820610636>3.0.co;2-8 3342383

[14] Kus LH, Shah M, Eski S, Walfish PG, Freeman JL. Thyroid cancer outcomes in Filipino patients. Arch Otolaryngol Head Neck Surg. 2010;136(2):138‐142. 10.1001/archoto.2009.206 20157058

[15] Nguyen M‐L, Hu J, Hastings KG, et al. Thyroid cancer mortality is higher in Filipinos in the United States: an analysis using national mortality records from 2003 through 2012. Cancer. 2017;123(24):4860‐4867. 10.1002/cncr.30958 28881423 PMC5716919

[16] California Cancer Registry . Accessed November 18, 2018. http://www.ccrcal.org/Inside_CCR/About_Us.shtml

[17] Percy C, Holten VV, Muir CS, eds. International Classification of Diseases for Oncology. 2nd ed. Geneva, Switzerland: World Health Organization; 1990.

[18] National Cancer Institute . SEER Extent of Disease‐‐1998. Codes and Coding Instructions. 3rd ed. Bethesda, MD: Cancer Statistics Branch, U.S. Department of Health and Human Services, NIH; 1998.

[19] Greene FL, Page DL, Fleming ID, et al. AJCC Cancer Staging Manual, 6th ed. New York, NY: Springer‐Verlag; 2002.

[20] Edge S, Byrd D, Compton C, Fritz A, Greene F, Trotti A, eds. AJCC Cancer Staging Manual. 7th ed. New York, NY: Springer‐Verlag; 2010.

[21] Yost K, Perkins C, Cohen R, Morris C, Wright W. Socioeconomic status and breast cancer incidence in California for different race/ethnic groups. Cancer Causes Control CCC. 2001;12(8):703‐711. 11562110 10.1023/a:1011240019516

[22] Spitz MR, Sider JG, Katz RL, Pollack ES, Newell GR. Ethnic patterns of thyroid cancer incidence in the United States, 1973–1981. Int J Cancer. 1988;42(4):549‐553. 10.1002/ijc.2910420413 3170027

[23] Espiritu GAM, Malana JT, Dumasis AJGV, Ang DC. High preponderance of BRAF V600E mutation in papillary thyroid carcinoma among Filipinos: a clinicopathologic study. J Glob Oncol. 2019;5:1‐6. 10.1200/JGO.18.00085 PMC6426509 30694737

[24] Navarro‐Locsin CG, Chang AMV, Daroy ML, Alfon AC, Andal JJ, Padua PF. Clinical and histopathological profile of BRAF V600E mutation in conventional papillary thyroid carcinoma in a Filipino population. Malays J Pathol. 2016;38(2):141‐148. 27568671

[25] United States Census Bureau . 2018 American community survey 5‐year data profile. Accessed November 4, 2020. https://data.census.gov/cedsci/table?t=Race%20and%20Ethnicity&g=0100000US_0400000US06&d=ACS%205‐Year%20Estimates%20Data%20Profiles&tid=ACSDP5Y2018.DP05&moe=false&hidePreview=true

